# Use of RNA interference to discover pathways involved in HIV infection and replication: cell lines tell many stories, primary cells might tell the truth

**DOI:** 10.1186/1742-4690-8-S2-P34

**Published:** 2011-10-03

**Authors:** Veronica lannucci, Alessia Landi, Jolien Vermeíre, Adele Mucci, Evelien Naessens, Hanne Vanderstraeten, Pieter Meuwissen, Julia Eekels, Ben Berkhout, Mostafa Bentahir, Bruno Verhasselt

**Affiliations:** 1 HIVlab, Department of Clinical Chemistry, Microbiology and Immunology, Ghent University, De Pintelaan 185, B9000Gent, Belgium; 2 Laboratory of Experimental Virology, Department of Medical Microbiology, Center for Infection and Immunity Amsterdam (CINIMA), Academic Medical Center of the University of Amsterdam, K3-110, Meibergdreef 15, 1105 AZ Amsterdam, the Netherlands; 3 Centre de Technologies MoléculairesAppliguées, Ecole de Santé Publigue, Brussels, Belgium

## 

Infection by Human Immunodeficiency Virus is difficult to treat thanks to its persistent viral reservoir and to its high rate of mutation that allows appearance of resistance to the available treatment. An approach to discover drugable targets is the identification of cellular partner proteins interacting with the virus during its life cycle.

We used RNAi technology to identify new HIV partners in the host cytoskeleton since it has been shown that cytoskeleton components and the irregulators have a role during several steps of HIV life cycle. By transducingseveral Tcell lines such as Jurkat CD4 CCR5, Jurkat E6-1 and SupT1 with lentiviral vectors expressings hRNA sequences, we silenced different target genes, members of pathways involved inactinrearrangement. By infection with HIV-NL4.3-eGFP reporter virus we evaluated HIV replication ratesin transducedcells. Surprisingly, the infection rate affected by the specific knock-down was dependent on the cell line used. lndeed, ashRNAtransduced in one cell line affected infection differently to what it did in another. Moreover, we observed that transduction on itself with a control vector expressinga scrambled shRNA sequence affected HIV infection rate in some but not all cell lines.

Therefore, to obtain relevant results in screening co-factors for HIV infection, we turned toprimary cells, the naturaltargets of the virus in vivo. We optimized combinedlentiviral transduction and HIV infection in cultured peripheral blood CD4+ lymphocytes. In this setting, transduction with scrambled shRNA expressing lentivirus did not affect HIV replication, providing us a platform to assay gene-knockdown likely to generate the most relevant information for natural HIV infection *invivo.*

